# Correction to “Whole-Cell Bioconversion of Renewable Biomasses-Related Aromatics to *cis,cis*-Muconic Acid”

**DOI:** 10.1021/acssuschemeng.3c01386

**Published:** 2023-03-30

**Authors:** Filippo Molinari, Loredano Pollegioni, Elena Rosini

The authors apologize that in the Abstract of their original article (https://pubs.acs.org/doi/full/10.1021/acssuschemeng.2c06534) there is a typing error for the amount of *cis,cis*-muconic acid (ccMA) produced by the wheat bran. When the optimized *E. coli* strain expressing all enzymes was used, ccMA was produced with a greater than 95% conversion yield starting from ferulic acid in 10 h following product isolation, corresponding to 2.2 mg of ccMA/g of wheat bran biomass (not 2.2 g).

